# Reintegration of cyber veterans: challenges and strategies for post-war transition

**DOI:** 10.3389/fpsyg.2024.1373245

**Published:** 2024-04-12

**Authors:** Toni Virtanen, Christina Lassfolk, Andrii Davydiuk, Mart Noorma

**Affiliations:** ^1^Finnish Defence Research Agency, Riihimäki, Finland; ^2^NATO Cooperative Cyber Defence Centre of Excellence (CCDCOE), Tallinn, Estonia; ^3^State Special Communications Service of Ukraine (SSSCIP), Kyiv, Ukraine; ^4^Pukhov Institute for Modelling in Energy Engineering, National Academy of Sciences of Ukraine (NAN Ukraine), Kyiv, Ukraine; ^5^Tartu Observatory, Faculty of Science and Technology, University of Tartu, Tartu, Tartu County, Estonia

**Keywords:** cyberpsychology, veteran adjustment difficulty, mental health, cyber veteran, volunteer fighters, post-war (re)integration

## Abstract

Battles in the cyber domain often lack visibility compared to the physical domain, which can lead to insufficient appreciation of its actors’ achievements by the general public and society. Nonetheless, it has become a crucial area of modern warfare in which cyber professionals defend the nation’s critical infrastructure, support other military operations and achieve military objectives that could not be attained in other domains. Although cyber combatants might not have as high a risk of injury or death as those on the front lines, they still can face traumatic events and suffer from the same types of issues after the war as any other veteran. Earlier studies on war veterans have shown they have elevated risks of mental health issues, substance use, social problems and financial difficulties which might push some individuals towards a path of crime. The cyber domain is also not limited by time or place, which has given rise to cyber volunteerism in which skilled individuals around the globe decide to assist their own country or friendly nations. This publication aims to identify the challenges cyber veterans might face when transitioning back to civilian life and develop appropriate strategies to facilitate their reintegration. It also raises the awareness of the hundreds of thousands of volunteers who can develop similar problems after the war. Without proper awareness, support and dialogue, some of these individuals can also drift towards harmful social implications such as cybercrime.

## Introduction

Along with the development of new technologies, military activities have shifted into new environments, creating a hybrid format of warfare in multi-domain operations. In the 21st century, cyberspace has become a new battleground (Kremer and Müller, 2013; Lonergan and Lonergan, 2023), where cyber domain is recognized as a separate domain for conducting military operations (NATO, 2016; Ministry of Defence, 2023). The significance of the cyber domain cannot be overstated as its impact can occur at the operational, tactical and strategic levels. During wartime, both military and civilian experts from defense agencies conduct cyber operations aimed at achieving military objectives. The effects of cyber operations could even surpass the results of actions on the battlefield, making the cyber domain a significant component in confrontations (Lee et al., 2016).

Measures in the cyber domain are also aimed at supporting information warfare and acting as a force multiplier for air, maritime, space and land operations (US FM 3-0 Operations, 2022). Given this, participants in cyber operations and cybersecurity experts are full-fledged contributors in combat operations and must be reintegrated into civilian life post-war like other veterans (D’Urso, 2015). Simultaneously, the high level of secrecy surrounding their activities and the lack of visibility of their achievements in the information space can elicit ambiguous attitudes toward their work among the civilian population and military personnel on the front line. At first glance, the risks of injury or death appear to be greater for soldiers on the battlefield, leading a significant portion of society to undervalue the efforts of cyber veterans compared to other veterans. However, many cybersecurity experts need to be positioned near the critical infrastructure they are defending which, despite international humanitarian law, can still be one of the main targets of enemy long-range strikes. Giving support to other combat operations might also require closer proximity to the front line exposing the cyber professionals to similar risks as other combatants on the battlefield.

Even without the hostilities of war, work-related stress can be high in cybersecurity professions (Nobles, 2022; Singh et al., 2023). When this is combined with the additional concerns of personal health and safety, atypical working conditions, high stakes in operations and possible exposure to traumatic events, they will endure considerable strain in their tasks. For their work, they might need to spend a considerable amount of time in the adversary’s networks, immersing themselves in hostile information environments and propaganda for bits of valuable intelligence. The emotional distancing from this will also require considerable stress endurance. From this, it can be concluded that during and after the war, appropriate psychosocial support and reintegration assistance for cyber veterans is needed. The terms reintegration, transition, readjustment and community integration are commonly used to describe the process of exiting the military and returning to the civilian world (Elnitsky and Blevins, 2017). In this paper, we will not make a distinction between the nuances of these terms and use them all to refer to the transitioning process of an active cyber combatant role to a civilian non-combatant role.

A separate challenge may arise from the distinction between enlisted or mobilized cyber combatants in the military, paramilitary cyber professionals and civilian cyber volunteers; whether the individuals not part of the country’s official armed forces can be called cyber veterans after a war. Nations can always prohibit paramilitary organizations outside the government or incorporate them under military command during a crisis when its members are considered part of the military forces. However, unlike military or paramilitary personnel under military command, individual cyber volunteers are much more difficult to identify and their personal contributions hard to evaluate. Without an official veteran status, they will probably fall through any psychosocial support programmes that might be provided. This question significantly deepens the problem of the reintegration of cyber warriors as there may be foreign cyber fighters, hacktivists, vigilantes, even minors and others that can be anywhere around the globe, where their actions would probably be considered as criminal activity by the regional authorities.

In this publication we review current literature on what type of reintegration challenges veterans face and consider wheter similar and novel types of challenges are expected for cyber veterans. In addition we explore some veteran support programmes and evaluate their fit for cyber veterans. The aim of this publication is threefold. First, to raise awareness of the global nature of cyber volunteers in modern conflicts and the potential social implications of these unseen cyber combatants. Second, to identify the challenges of reintegrating cyber veterans and third to explore appropriate strategies for the government to facilitate their return to civilian life.

## Methods

The intent of this study was not to conduct a systematic review as already preliminary searches indicated that research about cyber veterans, within the scope the authors had defined it, had not surfaced as a subject of robust academic research. This paper rather constitutes an explorative study of a potential new phenomenon. Providing an excursion and extrapolation from existing fields such as veteran studies and cyber warfare towards a related topic of cyber veterans.

The review proceeded roughly in two phases. Phase one focused on the regular veteran issues, reintegration challenges and transition support programmes from active service to civilian life providing the initial framework for the phenomenon. The second phase focused on the special characteristics of cyber warfare and the stressors cyber combatants face in their line of duty. Google Scholar served as search engine in a semi-systematic manner. Search terms were such as cyber, definition, package, reintegration, transition, veteran, warrior in various combinations. In overall, reachable, relevant articles in non-predatory publications with reasonable impact served as raw material for the present article. Several literature searches were conducted during the course of this study by each of the authors independently. These literature searches were periodically discussed during online meetings for their relevance and quality. The academic literature review was complemented with relevant legal and military doctrine documents and regular Google searches to deliver material for some illustrative case examples.

### Definition of a cyber veteran

According to the Oxford English Dictionary (2023), a veteran is a person who has had long experience in a particular field. However, many people associate the term with war veterans. A loose definition would therefore be everyone who serves in the military and survives will become a veteran (Kleykamp et al., 2021). Another way to define a veteran is when military personnel return from war and are discharged from military service they are assigned the label of veteran (Brown, 2011). However, nations differ in their requirements for achieving veteran status. This status is often further divided into war veterans, disabled veterans or overseas operations veterans, as their benefits may vary (38 USC, 2011; UK Public General Acts, 2021; UK Public General Act c48, 2023). In this paper, we define the term cyber veterans as individuals who have actively participated in the development, implementation and execution of national cyber capabilities to protect and secure the digital infrastructure and information assets during a time of war. This definition deliberately does not take a stand on the legislation and requirements of whether a person would receive a veteran status by their native country as this research is intended to address the potential issues on reintegration of cyber combatants after the conflict.

### The special case of cyber volunteers

Our definition of cyber veterans does not exclude non-military personnel participants in the war such as cyber volunteers although to avoid ambiguity, cyber volunteers are not to be mixed with military volunteers who enlist for service freely and are then part of the military. These cyber volunteers are skilled individuals who have decided to assist their own country or friendly nations (Ottis, 2010). A topical example comes in the wake of the 24 Feb 2022 escalation of the Russo-Ukrainian war as Russia attacked Ukraine. Ordinary citizens from Ukraine and other countries have become cyber volunteers. Although this type of co-production of cyber security is not new (Chang et al., 2018), this cyber vigilantism phenomenon is on a scope and scale not seen before (Soesanto, 2022). Their voluntary assistance involves supporting the state sector in cybersecurity, including providing computational resources, technical and software support and consultancy. Some individuals have also attempted to attack the resources of the enemy, prompting the Ukrainian government to respond with legal clarifications regarding such actions. The government continues to coordinate support from cyber volunteers to avoid any violations of Ukrainian and international legislation. However, as these individuals are collaborating by their own volition and ways, there is always a risk of exaggerated actions conducted by overzealous cyber volunteers. Currently, a significant portion of cyber volunteers has found the opportunity to officially collaborate with the government and this trend is growing. Ukraine is considering the formation of a cyber reservist unit in the armed forces as an additional resource in the event of massive aggression in the cyber domain (Tkachuk, 2023).

Cyber volunteers can also create an increased element of uncertainty in the post-war period. Well-prepared and cohesive teams of cyber professionals not controlled by the government could pose a significant threat to national security as their actions could provoke further escalation of the conflict. Additionally, risks can be caused by individual specialists which could arise from financial motives or disagreements with government decisions. After the war, dissatisfaction may emerge if the cyber volunteers feel their contributions are not recognized and their previous roles are diminished. Thus, to foster constructive dialogue between the government and cyber volunteers and to minimize the risks posed by them, it is essential to focus on social policies that recognize their significance, acknowledge specific individuals for their significant contributions to cyber defense, involve them in conferences and participate in the development and discussion of regulatory documents for the advancement of cybersecurity. It is also important to raise awareness among citizens about their contributions and to foster trust in cyber volunteers in society.

### General stressors in the cybersecurity profession

Working in the field of cyber security has been compared to working in a warlike environment (Brody, 2015; Singh et al., 2023). Cyberspace is contested at all times. Malign actors seek to degrade critical infrastructure, interfere with government services, extract intelligence, steal intellectual property and impede military activities (NATO, 2022). Cybersecurity professionals need to adapt to the fluctuating and rapidly evolving occupational requirements in an adversarial landscape. They provide a mission-critical service for ensuring operational security of armed forces and ensuring business continuity of private companies and government services (Paul and Dykstra, 2017). Offensive cyber operations can involve another set of demands where performance is highly dependent on speed and precision, not unlike the demands of fighter pilots or surgeons. Offensive cyber operators require a specialized set of skills and breadth of expertise from network fundamentals to adversarial thinking to strive in a high-risk high-reward environment (Dykstra and Paul, 2018).

Recent studies have indicated that work-related stress is high in the cybersecurity profession (Nobles, 2022; Singh et al., 2023). According to a 2021 CIISec State of Profession report, over half of cybersecurity professionals report having stress-related sleeping issues (Wilson, 2022). The VMware Global Incident Response Threat Report from 2022 confirms that this trend has not changed and 51% of cyber security professionals self-report having symptoms related to burnout and of that group, 65% have considered leaving the cybersecurity profession altogether (VMware, 2022). Some of these results can be explained by the global shortage of cybersecurity (CISA, 2020; De Zan and Di Franco, 2020; Coutinho et al., 2023). However, it seems that the working culture in cybersecurity tends to glorify working long hours as almost half of the CIISec report responders report working over 41 h a week and some even working up to 90 h a week (Wilson, 2022).

### Psychosocial issues related to potentially traumatic experiences

According to a recent meta-analysis of military personnel engaged in wars and conflicts between 1945 and 2022, 24% develop depression, 16% have anxiety-related issues and 21% suffer from Post-Traumatic Stress Disorder (PTSD) (Lim et al., 2022). Those who do not report experiencing such problems (25%) still sense difficulty in many essential areas of life such as difficulties in social and societal behavior, relationships, taking care of themselves, getting a job and feeling belonging, with injuries and suicidal thoughts (Elnitsky and Blevins, 2017). Potentially traumatic events often involve acute, high-strain situations which are not uncommon in combat situations or in a time of war. Encountering a direct attack or being in a combat situation has been associated with a significantly higher risk of PTSD and other psychological problems later in life (Hoge et al., 2004). PTSD can include symptoms such as hypervigilance, hyperarousal, intrusive images or thoughts, emotional numbing, difficulty trusting others, irritability or outburst of anger, heightened sense of vulnerability and trauma-related nightmares induced insomnia (WHO, 2021; APA, 2022). These symptoms can become crippling to the individual leading to withdrawal from social interactions and avoidance of everyday activities which could trigger symptoms and emotional distress.

From meta-analytic studies, demographic risk factors for the development of PTSD are the severity of the traumatic event, lack of social support, adverse childhood experiences and later life stress (Brewin et al., 2000). A perceived threat to life, dissociative experiences and intensity of negative emotions during the traumatic event were also found to be additional risk factors for developing PTSD (Ozer et al., 2003). Cognitive vulnerabilities such as negative attributional style, rumination, looming cognitive style and anxiety sensitivity have also been suggested to explain some individual differences for the sensitivity in developing PTSD (Elwood et al., 2009).

Research on veterans from peacekeeping operations has indicated that 2 to 8% of them suffer from some degree of posttraumatic stress symptoms (PTSD) in 3 years of post-deployment (Pearn, 2000). Previous assumptions were that the time immediately following deployment is the hardest time for veterans. However, there are indications that problems increase with time (Vogt et al., 2022). For most, potential psychological symptoms will rise a year after their deployment (Kaikkonen and Laukkala, 2016). However, for some, the symptoms have a later onset where they might present even years later after the crisis management deployment (Bonanno, 2004; Gray et al., 2004). Overall, military personnel seem to present PTSD symptoms later compared to civilians experiencing traumatic events (Andrews et al., 2007).

### Challenges in adjusting to civilian life after a war

The process for military personnel returning home to civilian life from deployment and life immediately after deployment can be highly stressful. Difficulties related to this transition are common and more than half of veterans suffer from some kind of psychological symptoms (Romaniuk et al., 2020). This transition often entails exchanging social networks by leaving one group of relationships and being required to establish and develop relationships with another group (Hopewell and Horton, 2012). Exclusive interaction with only the previous military community may prove counterproductive to reintegration (Herman and Yarwood, 2014). It has also been noted that the stronger the military identity the veteran holds, the more difficult the transition to civilian life tends to be (Adeoti et al., 2022; Ben-Shalom et al., 2023).

While some symptomatic behaviors such as being hypervigilant could even be considered to be adaptive in the battlefield, they can be very maladaptive for typical civilian life (Grupe et al., 2016). Survivors of a traumatic event might also feel responsible for the death or injury of others, even if they had no real influence in that situation (Tangney, 2002; Murray et al., 2021). Feelings of guilt have been linked to substance abuse, PTSD and depression symptoms for war veterans (Davis et al., 2023).

Post-deployment adjustment difficulties also tend to co-occur or lead to other life problems such as relationship problems or financial difficulties. It is known that mental health issues can lead to adjustment difficulties such as social problems, unemployment and financial problems which can even further be bolstered by co-occurring substance abuse (Romaniuk et al., 2020). On the other hand, veterans’ skills and experience might not be suitable for civilian professions causing financial difficulties, increasing life stress, and leading to adjustment difficulties while sensitizing the individuals towards mental health issues (Elbogen et al., 2012). Senior officers can expect to land a senior position in the civilian job market right away. However, while technical skills such as computing may transfer easily, non-technical skills like communication may need adaptation and acclimatization to the civilian corporate culture. The business world can also be afraid of potential mental problems, inflexibility, inadaptability, regimented individuals and awkward reactions to stressful situations which can make veterans feel stigmatized (Davis and Minnis, 2017; Quirke et al., 2021). Relationship dysfunction is also a common challenge faced by individuals with PTSD. This dysfunction can present in a variety of ways from emotional numbing to domestic violence (Walker, 2020). Regardless of diagnosis, financial well-being has been linked to post-deployment adjustment where veterans having the means to cover basic needs were less likely to have issues with post-deployment adjustment such as criminal arrests, homelessness, substance abuse, aggression or suicidal behavior (Elbogen et al., 2012). Another protecting factor has been linked to gratitude. Being the recipient of thankfulness, recognition and obtaining personal benefits from the society may have salutary effects on everyday functioning of war veterans. Dispositional gratitude predicted greater daily positive affect, percentage of pleasant days over the assessment period, daily intrinsically motivating activity, and daily self-esteem over and above effects attributable to PTSD severity and dispositional negative and positive affect in the PTSD group but not the non-PTSD group (Kashdan et al., 2006).

### Challenges related to cyber veteran integration

With their unique skillset and experience, cyber veterans will probably be sought after workforce, where their experiences can help to better understand malicious actors’ tactics, techniques and processes, improving organization’s ability to defend themselves from cybercrime. Cyber volunteers from various backgrounds can even alleviate the global shortage of cybersecurity professionals (CISA, 2020; De Zan and Di Franco, 2020; Coutinho et al., 2023). However, these skills and knowhow can be used to do harm as well as to do good. The path of cybercrime can simply look attractive because it promises more immediate financial benefits. Especially if the cyber veterans lack social support while they suffer from debilitating mental health issues making it difficult for them to find or keep work. Some might see cybercrime as their only option just to make ends meet or acquire the financial security necessary to concentrate on overcoming their issues.

Challenge, curiosity and status are often linked to hacker motivations (Chng et al., 2022). By the Social Learning Theory, crime can be a behavior that is learned just like any other behavior (Bandura and Walters, 1977). If the cyber veterans were accustomed to breaking into systems during the war, this type of activity, accepted in the time of war, has become a deeply learned behavior where the hacker mentality could even be part of their identity. If these skilled individuals are not directed towards beneficial activities such as red teaming, penetration testing or bug bounty programmes, some might traverse to the black hat activities just to find challenges for their highly tuned skills.

The most sinister outlook for cyber veterans can even lead to radicalization. PTSD-related symptoms such as emotional numbness, feelings of vulnerability and alienation can lead to reduced empathy and indifference to others (Nietlisbach and Maercker, 2009a,b). Other PTSD symptoms such as a tendency to withdraw social contact, irritability and outbursts of anger can ostracize and marginalize them from society even further (Nietlisbach and Maercker, 2009a,b). There is evidence that historical trauma can sensitize to conspiracy beliefs (Bilewicz, 2022). Research has shown that radicalization is driven by multiple causes. Wide-ranging grievances in everyday life can push individuals towards a more absolute way of thinking, while narrower political or religious pull factors attract certain types of ideology (Borum, 2011). These factors might start a vicious cycle where some cyber veteran individuals feel that society does not appreciate their war effort as much as other’s who fought with a rifle instead of a keyboard. These embittered individuals might drift towards extreme ideological groups where they could find a sense of community and comradeship. Doosje et al. (2016) has depicted the path to radicalization in three stages. Phase 1 is characterized by a sensitivity to a radical ideology. In Phase 2, the individual becomes a member of a radical group. Finally, in Phase 3, this person is ready to act on behalf of the group’s ideology. The threshold for the final Phase 3 could be lowered in the cyber domain as it is easier to mentally distance oneself from the possible victims (Leukfeldt and Yar, 2016).

### Supporting the transition of cyber veterans to civilian life

Similar support programmes that have proven effective for veterans overall should also be beneficial for cyber veterans. Veteran support programmes should provide timely and appropriate support, start as soon as possible, be affordable and sustainable and have an approach that borrows and adapts from existing structures (NATO STO TR-HFM-263, 2021). In addition, preventive programmes such as Psychological First Aid could also mitigate further problems later on (Prykhodko et al., 2021). These veteran support programmes should at least include some interactive programmes, retreats or professional services that help those veterans with PTSD and other mental trauma (Pearn, 2000). Many of the support programmes also include assistance in finding education or employment to achieve financial security and facilitate social support for the veterans (Kranke et al., 2019; NATO STO TR-HFM-263, 2021; Adeoti et al., 2022). Often these programmes include activities that extend to include the veteran’s family members and they can help in reconnecting and healing broken internal connections in the families (Tsybko, 2023). Younger veterans, would also benefit from support that offers interactive re-socialization and re-cultivation aid organized in educational institutions in connection with their studies (Pedersen and Wieser, 2021). Interventions witch focus on strengthening personal resilience and available support before and after exit seem to protect against stress and depression and enable psychosocial functioning (Pietrzak et al., 2010).

To identify those most in need of support and optimally focus the limited resources, pre- and post-departure screening methods in the form of short but efficient survey batteries are recommended (Romaniuk et al., 2020; Markowitz et al., 2023). Although, not sufficient predictors on their own, background information and life situations might also provide some insight on who is in direst need of support. For example, research has indicated that overall financial well-being (Elbogen et al., 2012), higher education, rank, age and previous exposure to adversity can protect a person against excessive negative consequences of their transition (Adeoti et al., 2022; Markowitz et al., 2023). Another preventive measure comes from voluntary or anticipated exit (Adeoti et al., 2022). In addition, the more in advance and concrete the planning for the next phase in life is, the better the chances for a successful transition (Kleykamp et al., 2021). Many veterans do not find satisfactory employment after service due to lack of a long-term plan (Kranke et al., 2019; Merritt, 2020; Adeoti et al., 2022). Thus, it is important that career planning support starts when soldiers enter service and that it helps them stay up-to-date on how military and civilian careers intertwine (Merritt, 2020).

Social support, defined as the perceived or tangible availability of social resources at one’s disposal, is among the key factors for successful reintegration (Cohen et al., 2001). Peer support groups have been shown to improve veteran’s reintegration and have beneficial mental health effects. Peer support groups often benefit from having members at various stages of their recovery, giving hope to those in their early stages and a feeling of accomplishment to those further on (Drebing et al., 2018). Special attention is required in assembling peer support groups for cyber veterans as the cyber domain and their experiences might be too technical for other war veterans to relate to.

Cyber veterans might have the skills and expertise to be a sought-after workforce. Their transitioning out of military service could also be expected to involve fewer challenges as their identities and professional profiles should be more aligned with civilian ones (Davis and Minnis, 2017). Veterans often report having a feeling of differentness where they feel separate from the civilians due to the perception that civilians do not understand veterans and what they have gone through, which is seen as a root cause for reintegration difficulties (Kranke et al., 2019). Clinging to veteran identity can also hinder reintegration (Adeoti et al., 2022; Ben-Shalom et al., 2023). Some individuals might therefore benefit from navigational support, aiding them in finding a route from their old identity to a new one (Herman and Yarwood, 2014). Veterans need to be encouraged to find new reference groups (Ben-Shalom et al., 2023) and focus on the many things they have in common with civilians (Kranke et al., 2019). While this process might appear easier for cyber veterans, as civilians working in cyber security should provide a relatable reference group for them, it is still crucial to provide enough navigational guidance for them, to nudge them towards more constructive reference groups. Some individuals might migrate towards undesirable reference groups such as hacktivists or even cyber-criminal groups.

Cyber volunteers will most likely not receive any legitimate veteran status which would make them eligible for state benefits and support programmes. It is, however, important to recognize that these individuals might also have similar difficulties in adapting back to their ordinary lives. In addition, the participating voluntary groups should acknowledge that they are responsible for their participants even after the conflict is over. These groups could seek discussions with Non-Governmental Organizations (NGOs) and professional practitioners who are already working with war victims and veterans for assistance. As an example, one framework could be taken from the National Psychological Association of Ukraine (NPA) for civilian wartime hotline support that currently works across 21 countries (Palii et al., 2023). While most of the volunteers are required to remain anonymous for their own safety, secure online peer support groups and information-sharing platforms could be developed. These could provide general information about education and working opportunities in cyber security, where to find professional help and act as a peer support forum, where former cyber volunteers could discuss and share their thoughts and feelings anonymously.

## Discussion

This paper raised awareness of the social implications of cyber volunteers during and after conflicts, discussed what difficulties cyber veterans might face in their reintegration and explored the types of support programmes that should be developed to benefit their transition. As the cyber domain lacks similar visibility to the physical domain, it requires more effort from the media and societies to recognize the contribution of the cyber professionals, with and without uniform, as recognition and thankfulness have been shown to have salutary effects on the everyday functioning of veterans (Kashdan et al., 2006).

Especially for a mobilized army, most of the combatants will return to their civilian occupations after the war, while some might remain in the military. This transition requires adjusting and exchange of social groups (Hopewell and Horton, 2012). This process can lead to feelings of differentness, where veterans perceive their experiences in the war are not well understood by the civilians (Kranke et al., 2019). These feelings can be alleviated by peer support groups (Drebing et al., 2018). However, cyber veterans might be at a disadvantage as the cyber domain can be so technical that other war veterans might have difficulties relating to their experiences, while cyber volunteers are forced to remain anonymous for their own safety.

Reintegration difficulties tend also to co-occur or lead to other life problems such as relationship problems, financial difficulties, mental health issues and substance abuse. Financial well-being has been recognized as one of the key protecting factors for reintegration (Elbogen et al., 2012). With their unique skills and experience, cyber veterans will probably be a sought-after workforce. However, these skills and know-how can be used to do harm and to do good. The path of cybercrime can simply look attractive because it promises more immediate financial benefits. Especially in the global army of cyber volunteers, who already might have become accustomed to breaking the law in their native country for a cause they believe in, some individuals may drift towards criminal activity as implied by the Social Learning Theory (Bandura and Walters, 1977). To prevent this type of chain of events, more public discussion and dialogue with the cyber volunteers is needed. Many of them will be obliged to remain anonymous for their own safety and possible legal consequences by the regional authorities of their native country.

The notable limitation of this study is the lack of systematic review process. Applying a more rigid review methodology, would have created a better stepping stone for further research. Several important aspects of veteran integration, such as gender issues were also omitted to keep the text somewhat clear and concise. Future research directions for exploring this novel phenomenon would be to approach military and paramilitary cyber professionals to better understand what difficulties they have had or are expecting to face with their transition to civilian life.

## Author contributions

TV: Writing – original draft, Writing – review & editing. CL: Writing – original draft, Writing – review & editing. AD: Writing – original draft, Writing – review & editing. MN: Conceptualization, Supervision, Writing – original draft, Writing – review & editing.
